# 

**Published:** 1891-04

**Authors:** 


CONTENTS.

Vol. 38. No. 4.

| | PAGE. |
|---|---|
| The Hygiene of the Home | 73 |
| A Fact Stranger than Fiction | 74 |
| Short Lived Beauty | 77 |
| Rules for Good Health | 79 |
| The Finger Nails | 79 |
| Candies and Sweets | 80 |
| The Bath | 81 |
| Liver Spots | 81 |
| Marriage with Drunkards | 82 |
| Cold Food in Winter | 82 |
| The Value of Sleep for Women | 83 |
| Year-Old Babies | 84 |
| Oysters as Food | 85 |
| Managing the Little Ones | 86 |
| Gas Poisoning Hints | 87 |
| How to Treat One in a Faint | 88 |
| A Veteran's Manner of Life | 88 |
| Miscellaneous | 91 |
| Small Things of Great Account | 93 |
| Literary | 94 |
| The Costliest Gift | 96 |

INDEX TO ADVERTISEMENTS OF HALF A PAGE AND OVER.

| | PAGE. |
|---|---|
| Horsford's Acid Phosphate | I |
| Lydia E. Pinkham Med. Co. | II |
| Geo. P. Rowell & Co | IV |
| Hall's Journal Premiums | VI |
| Stonington Line | VII |
| Ruckel & Hendel | IX |
| Ayer's Sarsaparilla | IX |
| Horse and Stable Pub. Co | XI |
| Aerated Oxygen Comp'nd Co. | XII |
| Herba Vita | XIII |
| Fowler & Wells Co | XIV |
| J. H. Bunnell & Co | XVI |
| Turf, Field & Farm Ass'n | XVII |
| Royal Baking Powder | XIX |
| I. S. Johnson & Co | XIX |

